# Predicting Effortful Control at 3 Years of Age from Measures of Attention and Home Environment in Infancy: A Machine Learning Approach

**DOI:** 10.3390/children10060982

**Published:** 2023-05-31

**Authors:** Mariel F. Musso, Sebastián Moyano, Josué Rico-Picó, Ángela Conejero, M. Ángeles Ballesteros-Duperón, Eduardo C. Cascallar, M. Rosario Rueda

**Affiliations:** 1 Department of Experimental Psychology, University of Granada, 18071 Granada, Spain; mariel.musso@ugr.es (M.F.M.); smoyano@ugr.es (S.M.); rpicoj@ugr.es (J.R.-P.); 2 Interdisciplinary Center for Research in Mathematical and Experimental Psychology (CIIPME), National Council for Scientific and Technical Research (CONICET), Ciudad Autónoma de Buenos Aires 1040, Argentina; 3 Department of Psychology, Faculty of Health Sciences, Universidad Argentina de la Empresa (UADE), Ciudad Autónoma de Buenos Aires 1073, Argentina; 4 Mind, Brain and Behavior Research Center, University of Granada, 18071 Granada, Spain; angelaconejero@ugr.es (Á.C.); maballes@ugr.es (M.Á.B.-D.); 5 Department of Educational and Developmental Psychology, University of Granada, 18071 Granada, Spain; 6 Department of Psychobiology, University of Granada, 18071 Granada, Spain; 7 Faculty of Psychology and Educational Sciences, KU Leuven, 3000 Leuven, Belgium; cascallar@msn.com

**Keywords:** effortful control, self-regulation, attention, artificial neural networks, prediction, machine learning

## Abstract

Effortful control (EC) is a dimension of temperament that encompass individual differences in self-regulation and the control of reactivity. Much research suggests that EC has a strong foundation on the development of executive attention, but increasing evidence also shows a significant contribution of the rearing environment to individual differences in EC. The aim of the current study was to predict the development of EC at 36 months of age from early attentional and environmental measures taken in infancy using a machine learning approach. A sample of 78 infants participated in a longitudinal study running three waves of data collection at 6, 9, and 36 months of age. Attentional tasks were administered at 6 months of age, with two additional measures (i.e., one attentional measure and another self-restraint measure) being collected at 9 months of age. Parents reported household environment variables during wave 1, and their child’s EC at 36 months. A machine-learning algorithm was implemented to identify children with low EC scores at 36 months of age. An “attention only” model showed greater predictive sensitivity than the “environmental only” model. However, a model including both attentional and environmental variables was able to classify the groups (Low-EC vs. Average-to-High EC) with 100% accuracy. Sensitivity analyses indicate that socio-economic variables together with attention control processes at 6 months, and self-restraint capacity at 9 months, are the most important predictors of EC. Results suggest a foundational role of executive attention processes in the development of EC in complex interactions with household environments and provide a new tool to identify early markers of socio-emotional regulation development.

## 1. Introduction

### 1.1. Importance of Self-Regulation during Infancy and Life Outcomes

Self-regulation in childhood has been related to a diversity of concurrent and subsequent outcomes in adolescence and adulthood [1]. A broad definition of self-regulation commonly adopted in the literature refers to the ability to control impulses and to adapt thoughts, emotions, and behaviors [2]. Self-regulation has been discussed in the literature related to different constructs and cognitive processes. One broad construct related to self-regulation is executive functioning, which encompasses different cognitive processes such as inhibition, working memory, and shifting [3]. Inhibitory control is a specific self-regulatory skill defined as the inhibition of prepotent thoughts in order to allow a subdominant action (e.g., [4]). A series of meta-analyses have found a positive association between inhibitory control and academic performance in children from 3–6 years old [5] and intelligence in children under 12 years old [6]. In addition, recent meta-analyses have shown a negative association between childhood self-regulation and disruptive and aggressive behavior (externalizing problems) and negative emotions related to depression, anxiety, and suicidal thoughts (internalizing problems) [6,7]. Other meta-analyses have found a negative correlation between self-regulation and different victimization behaviors (e.g., online bullying) [8]. A recent meta-analysis including 150 empirical studies, comprising cross-sectional and longitudinal studies in childhood from different countries [2], also supported the previous findings. Moreover, the positive effects of childhood self-regulation have been shown on a variety of important outcomes in later life such as mental and physical health and healthy living [2]. High self-regulation at preschool age has been related to higher performance in mathematics, literacy, and vocabulary, and to lower peer victimization, disruptive behavior, and negative emotions in the first years of primary school [2] and childhood self-control predicts important adult life outcomes and behaviors, such as physical health, substance abuse, personal financial situation, and criminal offenses [9].

A broad neurobiological model of self-regulation based on the theoretical framework of temperament [10] states that bottom-up reactivity and top-down regulation processes interact during development [11,12]. From this perspective, effortful control (EC) is the temperamental factor more closely related to self-regulatory abilities, being defined as the ability to apply volitional control over reactive systems either of approach or withdrawal [13]. Different behavioral traits are targeted by EC, such as inhibitory control, attentional focusing and shifting, or perceptual sensitivity, among others [14]. In general, EC is the dimension of temperament that captures individual differences in children’s self-regulatory skills with a strong attentional foundation [15], especially in executive attention processes [16,17]. As EC is an important nexus between self-regulation and attention control abilities, we aim to employ EC as a proxy for self-regulatory skills, while considering the predictive power of early attention control and home environment factors.

### 1.2. Attention as the Foundational Basis for Self-Regulated Behavior

In general, attention emerges as a key asset for the development of top-down control. Research seems to support this notion, with attention being at the basis of self-regulation [18], sharing common brain structures essential for both volitional control of behavior and attention [19].

Development takes place in constant interaction with the environment. Infants continuously receive information (input) from its context, while generating responses (output) in consequence. However, as the cognitive system is of limited capacity, a mechanism has evolved to regulate the input source of information as well as the course of thoughts and actions. According to Posner’s model of attention, various networks of brain areas are involved in three functions of attention. The locus coeruleus, a region located in the brainstem, together with cortical areas of the frontal cortex, is involved in maintaining the adequate level of activation necessary to respond to stimulation. In addition, a circuit of brain areas in the parietal and frontal cortex works to select and prioritize the processing of relevant information (selective attention). Finally, a circuit of brain regions with a main node in the anterior cingulate cortex is involved in regulating thoughts and actions in relation to internal given goals [20]. Attention is therefore related to goal-driven behavior, being the foundational mechanism for the volitional control of thoughts, emotions, and actions [18,21]. 

According to its role in self-regulation, attentional abilities have been found to be related to the development of children’s cognitive and academic skills and socio-emotional adjustment [1], as well as life outcomes in adulthood [9]. Increases in attention control enable infants to implement self-controlled strategies to down-regulate emotional states and behavioral reactions [22]. In the first half of the first year of life, attention control can be used to disengage and shift attention away from distressful events or stimulation. In this sense, attention serves the purpose of downregulating infants’ behavioral and emotional reactivity [23,24]. The previous literature suggests a consistent positive association between attention disengagement and infants’ EC (i.e., soothability and regulation of distress) between 4 and 12 months of age [25,26,27]. At the same time, other aspects of attention control during infancy are also associated with self-regulation. For instance, the Visual Sequence Learning (VSL) task has been previously used to measure correct anticipatory looking as a proxy for endogenous attention control in a sample of 6-to 7-month-old infants. Interestingly, infants with more correct anticipations displayed longer durations of self-soothing behavior to down-regulate reactivity after being presented with a distressful mask [28]. 

Although these results suggest a concurrent association between infants’ attention and self-regulatory skills, infant research has also found longitudinal relations. For instance, a previous study [29] measured infants’ focused attention at 9 months of age, while at 22 months they were administered an EC battery, including self-restraint and response inhibition tasks. Results indicated that the higher the focused attention during infancy, the better the self-regulatory abilities would be during toddlerhood. Similarly, infants with higher sustained attention at 10 months of age were also found to show a better ability to self-regulate frustration at 36 months when solving a challenging puzzle [30]. Following this notion, recent studies have targeted infants’ early attention control through fixation durations. Results highlight a positive association, with longer fixations between 7 and 11 months of age predicting higher EC during toddlerhood [31] and early childhood [32]. Additionally, the higher the duration of fixations, the fewer behavioral problems there were during early childhood [33]. 

### 1.3. Impact of the Rearing Environment on Self-Regulatory Abilities

Increasing evidence is showing that the early experiences of the child influence early stages of cognitive development [34]. For instance, caregiving factors such as the early home environment, family characteristics, or parenting styles appear to influence the development of self-regulation in the first years of life [12,35]. A large body of cross-sectional and longitudinal studies has shown the family’s socioeconomic status to influence early attention and self-regulation development [36,37,38,39].

A family’s socioeconomic status (SES) is one of several factors that are known to define infants’ environment. Children from low-SES backgrounds are more likely to be exposed to restricted economic and educational resources necessary to support children’s optimal development [40]. The effects of prolonged exposure to a low-SES background have been found to alter the developmental trajectory of self-regulation in childhood [11,41,42] and in adulthood [42,43]. The duration of exposure to adversity and how early it is experienced seem to be critical factors. In a longitudinal study, Raver et al. [44] measured the years of infants’ exposure to a low-SES environment from infancy to childhood. The years of exposure significantly contributed to the prediction of children’s emotion regulation at 58 months of age. Similarly, early exposure to low-SES environments in infancy is also predictive of lower EC and emotion regulation at 60 months of age [12].

Interestingly, in a previously mentioned study [44], CHAOS was found to contribute to the prediction of children’s self-regulatory abilities. The measure of CHAOS is characterized by high levels of an unstructured environment combined with low levels of predictability and established routines, together leading to high environmental confusion [45]. The CHAOS construct offers a different level of analysis on the impact of the environment on children’s development. It captures different environmental characteristics than SES [46,47] and is likely to be distributed across different SES backgrounds [48]. A recent meta-analysis covering the period from 2 to 17 years of age shows that the effects of home chaos are widely spread across development [49]. During early childhood, CHAOS at 30 months of age is negatively associated with self-regulatory abilities at 30, 42, and 54 months [50]. Similarly, household disorganization was measured during the first three years of children’s lives [51]. Although no direct effect of home chaos over self-regulation was found, chaotic home environments indirectly impacted self-regulatory abilities through parenting behaviors and children’s EF at 36 months. 

Apart from SES and CHAOS, maternal depressive symptomatology is of special relevance during the perinatal period. With a prevalence of almost 12% [52], maternal depression shows a negative impact on the development of children’s self-regulation. Maternal depressive symptomatology is likely to reduce infants’ stimulation from mother-child interactions [53,54]. In addition, it increases exposure to environmental stressors that could hinder early brain and cognitive development [55]. Moderate levels of maternal depression from birth up to the second year of life have been reported to have a negative impact on behavioral and emotional regulation during early childhood [56]. Similar results are reported at older ages, with maternal depression during toddlerhood predicting more behavioral problems during toddlerhood [57] and lower EFs during childhood [58,59]. We have seen in the previous paragraphs how attention and the infants’ environment impact later self-regulatory abilities. But how do these two factors interact to predict development?

### 1.4. Using Machine-Learning to Understand the Multiplicity of Factors Contributing to the Development of Self-Regulation

The development of self-regulation is a complex process. As discussed in previous sections, several intrinsic (e.g., attention) and extrinsic factors (e.g., environment) to the infant are known to impact their developmental trajectory. Therefore, the development of self-regulation is very likely to involve dynamic processes with critical periods from birth to adulthood [60]. Several studies have shown that physical, neural, and cognitive systems interplay through a hierarchical cascade process, from which emerges a gradual control during childhood [61,62]. However, most of these studies have used classical approaches which do not simultaneously examine the complexity of the interrelationships among these multiple developmental factors. These approaches have the usual parametric constraints of traditional statistical methods, and they do not achieve very accurate predictions or classifications [63]. Therefore, a more robust and precise methodology based on machine learning algorithms is needed in order to address the complex nature of the early development of self-regulatory behaviors. These types of methodologies have been developed and applied during the last decade in different fields, such as education and health, with predictive and classificatory purposes [64,65]. 

The aim of this study was to examine whether the level of development of EC at 3 years of age could be predicted from early attentional and environmental measures taken in infancy using a machine learning methodology such as artificial neural networks (ANN). In addition, this study aims to identify patterns of individual and environmental variables at 6 to 9 months of age that could allow an accurate predictive classification of self-regulatory difficulties (i.e., low-EC) at 3 years of age.

## 2. Materials and Methods

### 2.1. Participants

Families were provided full in-person information about the purpose of the study and those contacted by researchers during informative visits at the Maternity Hospital of Granada were given a leaflet. As some families contacted the lab via telephone after seeing informative posters in public health centers, researchers provided full detailed information about the study during the call and sent a study leaflet through email. From a pool of 216 families that gave their initial consent to participate, as well as their contact details, 160 families agreed to come to the Developmental Cognitive Neuroscience Lab when their infants were 6 months of age. Infants were included in the study if they fulfilled the following criteria: (1) weight at birth was higher than 2500 g, (2) they were born at term (37 weeks at least), and (3) they did not present with any medical condition at birth. From the initial sample, *n* = 18 did not meet these inclusion criteria (*n* = 6 criteria 1; *n* = 10 criteria 2; *n* = 2 criteria 3). At 6 months, the final sample was composed of 142 infants, 122 at 9 months and 92 at 36 months (see Table 1 for descriptive statistics). 

For the neural network analyses, only those children with full data across the three waves were included, so the final sample for these analyses included a total of 78 children.

### 2.2. Apparatus

An EyeLink 1000 Plus [66] corneal-reflection eye-tracker was employed to collect gaze information during the eye-tracking tasks, with a sampling rate of 500 Hz and 0.01° of spatial resolution using a lens of 16 mm and an illuminator of 890 nm. Task presentation was controlled through Experiment Builder software [67], being presented in an LG 24M37H-B 24-inch LED monitor with a native resolution of 1920 × 1080 pixels (52 × 30 cm). A five-point child-friendly calibration procedure was administered before task initiation using looming colorful shapes (1.97° × 1.97° of visual angle) accompanied by melodic sounds. Calibration points were manually presented in the corners and center of the screen and were repeated until the experimenter reached a satisfactory calibration result. 

A sample report with raw gaze data was obtained for each participant using Data Viewer [68]. Raw data was fed into the Python implementation of the identification using a two-means clustering (I2MC) algorithm [69] to parse fixations with a minimum fixation duration of 100 ms. The I2MC automatic algorithm was developed to deal offline with noisy data when periods of data loss could occur. It has been found to be less affected by differences in precision between 0–2° of RMS-s2s deviations, which is rarely to be found over 3° in infant research [69]. Data reduction was performed using custom written Python 3 code once fixations were parsed.

### 2.3. Experimental Tasks

#### 2.3.1. Gap-Overlap Task

**Figure 1:**
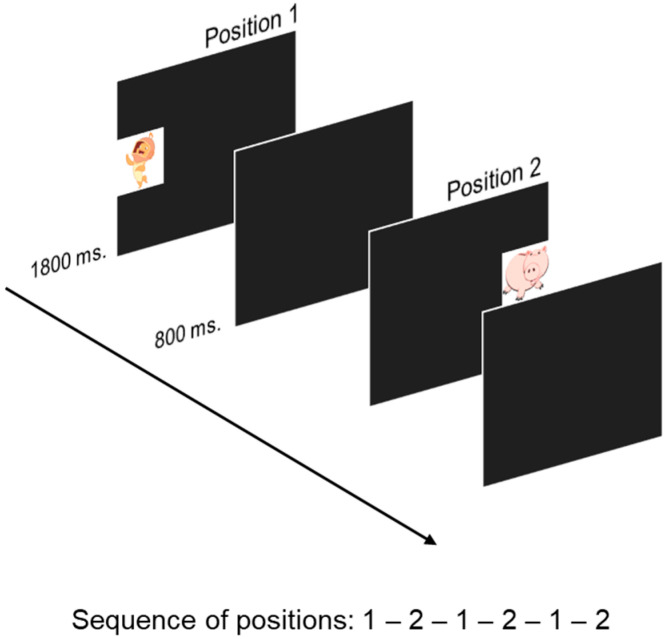
Procedure for the Visual Sequence Learning task for 6-month-old infants.

For the gap-overlap task, we considered only the overlap and gap conditions (see [70]). At the beginning of each trial, an animated stimulus was presented in the center of the screen (10.31° × 10.31°). When the experimenter observed a fixation on the stimulus, he/she pressed a key to continue with the trial. In the overlap condition, the peripheral target (6.76° × 6.76°) was displayed along with the central stimulus, with both remaining on screen until the end of the trial. For the gap condition, the peripheral target was displayed after a 200 ms temporal gap interval that was initiated after the offset of the central stimulus [71]. Peripheral targets were presented for 1000 ms on the left or right side of the screen (13.11° of eccentricity to the nearest edge of the stimulus; see Figure 1). Forty-eight trials were administered in a pseudo-randomized order, with no more than two consecutive trials of the same condition being sequentially repeated. The median of the SLs (mdSL) was computed for each participant for the overlap and gap conditions. Additional information concerning the analysis of the task can be found in Supplementary Materials (in Section S1.1).

#### 2.3.2. Visual Sequence Learning (VSL)

An adapted version of the original VSL task [72] was developed to be used with 6-month-olds. Similar to the expectation paradigm [73], we presented stimuli in the central left (position 1; 14.93° × 9.46° of eccentricity) and central right side (position 2; 14.93° × 9.46° of eccentricity) of the screen in a fixed sequence (1-2; see Figure 1). Infants were presented with a total of 24 trials. The first 4 trials were considered practice trials (16.6% of total trials), while the remaining 20 trials were considered experimental. We computed the percentage of stimulus fixation over the total number of experimental trials, as well as the proportion of reactive looks and correct anticipations based on total stimulus fixations. Additional information concerning the analysis of the task can be found in Supplementary Materials (Section S1.2).

**Figure 2:**
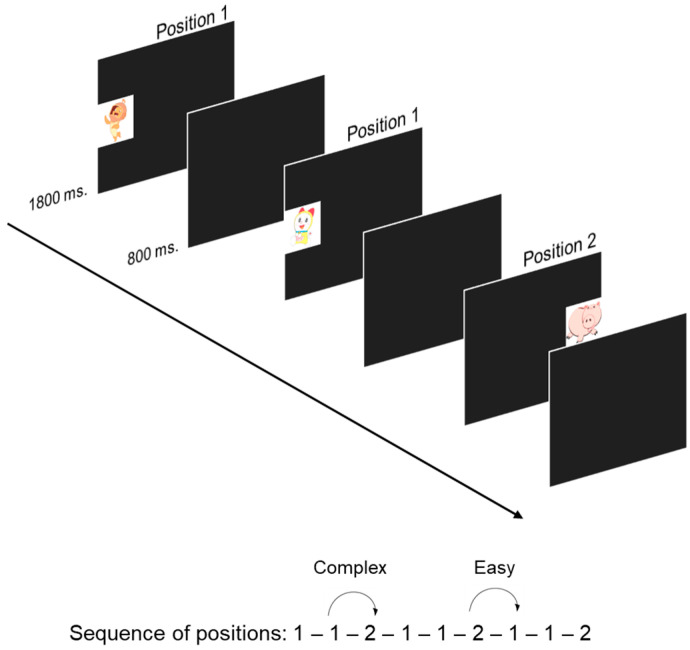
Procedure for the Visual Sequence Learning task for 9-month-old infants.

A modification of the sequence was introduced in the 9 months version of the task in order to introduce a distinction between easy (unambiguous; context-free) and complex (ambiguous; context-dependent) trials. Again, stimuli were presented in the central left (position 1) and central right side (position 2) of the screen in a fixed sequence (1-1-2 [74]; see Figure 2). Infants were presented a total of 48 trials, with the initial 9 trials being considered as practice trials (18.75% of total trials), while the remaining 39 trials were considered experimental. In this version, position 1 was repeated two times in a row before position 2. This particular sequence (1-1-2) allows us to distinguish between anticipations in which the next stimulus position could be unambiguously predicted (i.e., position 2 is always followed by position 1) or ambiguously predicted (i.e., position 1 could be followed by position 1 if it is the first occurrence in the sequence, or by position 2 if it is the second). For ambiguous trials, infants would be required to engage in context monitoring processes in order to keep track of the previous position to the current one to correctly anticipate the next stimulus location.

Again, we computed the percentage of stimulus fixations over the total number of experimental trials and the proportion of reactive looks based on the infant’s total stimulus fixations. We also computed the proportion of correct anticipations in complex trials based on total anticipations (both correct and incorrect anticipations) for complex trials [14]. Additional information concerning the analysis of the task can be found in Supplementary Materials (Section S1.2).

#### 2.3.3. Switching Task

**Figure 3:**
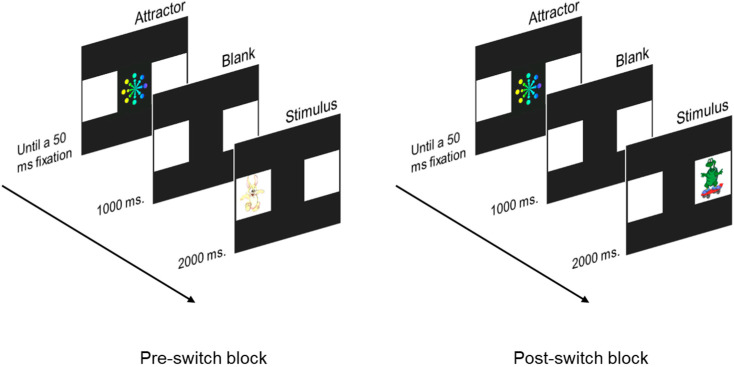
Procedure for the switching task. Locations of stimulus presentation in the pre-switch block were counterbalanced between participants.

We employed an adaptation of an attention-switching task to evaluate attention flexibility at 6 months of age [75] (see Figure 3). Two white boxes (15° × 15°) were presented at either side of the screen at 9.66° eccentricity to the nearest edge of the box over a black background during the entire trial. Each trial started with a colorful animated attention attractor in the center of the screen coupled with music. After a 50 ms fixation on the attractor, an anticipatory period was introduced, displaying only the two white empty boxes for 1000 ms. Finally, an animated cartoon coupled with a funny sound was presented for 2000 ms in one of the boxes. The task comprises two blocks. In the first block (pre-switch), the same stimulus was always presented on the same box (rewarded location) for a maximum of 18 consecutive trials. In the next block (post-switch), a different stimulus was presented on the opposite box (non-rewarded location in the pre-switch block) for twelve consecutive trials. A minimum of 3 correct anticipations were required before trial 18 in the pre-switch block in order to move to the post-switch block. This was required in order to be certain that the infant generated an expectation of the stimulus presentation side to fairly measure perseverative errors during the post-switch block. Both stimulus location and identity were counterbalanced between participants. The proportion of perseverative anticipations in the post-switch block was computed over the number of total anticipations (both correct and incorrect anticipations) [14] as a measure of attentional flexibility. Additional information concerning the analysis of the task can be found in Supplementary Materials (Section S1.3).

#### 2.3.4. Toy Prohibition Task

At 9 months of age, infants we administered the toy prohibition task. We followed the same procedure applied by Hendry and colleagues [76]. The caregiver and infant were seated in front of a table facing the experimenter. The entire procedure was recorded by two cameras, one from the infant’s side and another from the front. The infants’ latency to touch the glitter wand was coded offline by two independent coders. Infants that did not touch the toy before the experimenter encouraged the infant were assigned a latency of 30 s. Intraclass correlation coefficient (ICC) for single measures indicated excellent reliability (ICC = 0.99, *p* < 0.01). Additional information about the procedure of the task can be found in Supplementary Materials (Section S1.4).

### 2.4. Questionnaires

#### 2.4.1. Socioeconomic Status

Parents were asked about their professional occupation and family’s income at 6 months. Education level was scored from 1 (No studies) to 7 (Postgraduate studies). Likewise, professional occupation was rated following the National Classification of Occupations (CNO-11) of the National Institute of Statistics of Spain (INE) from 0 (Unemployed) to 9 (manager). Mean scores of parental education and occupation were computed as the average of the mother’s and father’s education and occupation level, respectively. In addition, an income-to-needs ratio was computed by dividing the family’s annual income by the official poverty threshold provided by the INE based on the number of members in the family unit. A general SES index was calculated by averaging the z-scores of the three socioeconomic aspects (mean parental education, mean parental occupation, and income-to-needs ratio).

#### 2.4.2. Confusion, Hubbub, and Order Scale (CHAOS)

A Spanish version of the CHAOS scale [45], previously adapted to the Spanish language [77], was completed by parents at 6 months of age to measure the level of confusion and household disorganization. Parents reported their level of agreement with different statements describing the organization, environment, and family routines at home through a six-point Likert scale (15 items, *α* = 0.87) ranging from 1 (Completely agree) to 6 (Completely disagree). A total score of chaos was computed by adding the scores for each item. The higher the score, the higher the reported level of chaos at home.

#### 2.4.3. Beck Depression Inventory (BDI)

The Spanish version of the Beck’s Depression Inventory (BDI-II; [78]) was employed to measure maternal depressive attitudes and symptoms at 6 months of age. The BDI-II is a 21-item self-reported inventory completed by mothers reporting how they felt in the last two weeks concerning different depressive symptoms. Answers were provided using a Likert scale from 0 to 3. The inventory showed an internal consistency of *α* = 0.88. A total score was calculated by adding the scores of the 21 items, with a higher score indicating higher depressive symptomatology.

#### 2.4.4. Children’s Behavior Questionnaire (CBQ)

At 36 months, parents completed the Spanish short version of the Children’s Behavior Questionnaire (CBQ; [79]) to measure children’s temperamental effortful control. Parents completed 94 items concerning their children’s behavior in different situations using a Likert scale from 1 (Extremely false) to 7 (Extremely true). Cronbach’s alpha for the CBQ scale and EC subscale were 0.87 and 0.74, respectively.

### 2.5. Procedure

Families were received in the Developmental Cognitive Neuroscience Lab located in the Mind, Brain and Behavior Research Center. Parents/legal guardians were given detailed information about the session and were required to sign informed consent while giving the infant time to feel comfortable with researchers. 

For the 6- and 9-month sessions, once parents/legal guardians and infants were ready, they were guided to the eye-tracking room to complete three eye-tracking tasks in a fixed order: starting with the switching task, followed by the VSL, and ending with the gap-overlap task. Infants were placed in a highchair with a head support pillow at approximately 60 cm from the monitor. Parents were seated behind the highchair to avoid infants being distracted. If infants showed inattention or fussiness, they were seated on her/his caregiver’s lap. Parents were asked to remain silent and avoid interacting with the infant during the entire procedure. Researchers controlled the administration of experimental tasks from an adjacent room, monitoring the infant’s behavior through a webcam camouflaged next to the eye-tracker lens. If needed, a short break was introduced between tasks, initiating a new calibration procedure if the task was interrupted. Once the eye-tracking procedure was finished, 6-month-old infants completed an EEG protocol, while at 9 months, the EEG protocol was preceded by the toy prohibition and other behavioral tasks that will not be presented in the current paper. At the end of the session, parents were informed about and sent questionnaires to be completed online at home. At 36 months of age, parents were contacted to complete the CBQ online. The present research is part of a larger longitudinal study in which additional measures were taken in other sessions. The study was approved by the Ethics Board of the University of Granada (Refs. 488/SEIH/2018 & 2536/CEIH/2021) following the Declaration of Helsinki. Participation in the current research was voluntary and legal guardians gave written consent before participating. Families were given an EUR 10 voucher for educational toys in compensation for their time at the 6- and 9-month sessions. For the 36-month-old session, families received an EUR 25 voucher.

### 2.6. Analysis Procedure

We implemented a multilayer perceptron ANN with a backpropagation algorithm to identify children with low (percentile 33 or below) vs. moderate/high (above percentile 33) EC scores at the age of 3 years. The 1/3 vs. 2/3 division was chosen in order to have sufficient cases for both training and testing the ANN predictive algorithm given the relatively small sample size of the study. The ANNs used have a structure of three or more layers: (1) the input layer including the predictors, (2) the hidden layer that represents the interactions between input and output, and the output layer that refers to the dependent variable, in this case, a classification between children with low EC vs. moderate/high EC at 3 years of age [80,81]. 

Three different ANN were developed for the classification of each child belonging to the lowest 33% of EC or not. The first one only involved attentional variables at 6 and 9 months (see Table 2). The second ANN only included environmental factors at 6 months (see Table 2), and the third one introduced both attentional and environmental variables. 

We followed a systematic procedure for the implementation and evaluation of the ANN suggested by the literature [82]. The available data set was randomly split into a training (70%) and testing set (30%) for each ANN. A 70% split was used in the training set in order to include a set of cases representing most of the patterns expected to be present in the data (patterns represented by the vector of information on the input variables for each case).

For the training of each ANN, the online learning method was selected, in which ANN learns by examining each individual case. This method is able to track small changes, and it is the most widely used supervised learning method for solving classification problems [83]. The implementation of a backpropagation algorithm follows two phases. In the forward phase, the predictive weights are generated and the input signal is transferred through the layers until the output classification is generated. The backward phase starts with the generation of an error signal given a correct or incorrect prediction by comparing the obtained output with the expected value. The error signal is back-propagated layer by layer and ANN adjusts the previous weights, minimizing the error in each cycle until one or more of the stopping criteria have been reached. Gradient descent was chosen in this study as an optimization function to minimize the error from the mean squared error function. The activation functions chosen were a hyperbolic tangent function as a transfer function of the hidden layer because it allows the ANN to identify nonlinear and complex relationships between the predictors [81]; and sigmoid and softmax functions as transfer functions for the output layer, given that they maximize the classification for dual and multiclass sets, respectively. 

During the training phase, several models were tested for each ANN, adjusting systematically the learning rate and the momentum parameters. The learning rate modifies the values of the weights in each iteration, and the momentum adds a fraction of the prior weight change to the present weight change thus increasing the speed of the learning process [84]. Initial learning rate values were: 0.6; 0.4, 0.8, 0.04, 0.1, 0.01, 0.001, and 0.0004. The following momentum values were used: 0.5, 0.7, 0.9, 1.2, and 1.5. Finally, the two models that achieved the best accuracy for both target and moderate/high EC groups in the testing phase were selected for each one of the ANNs and an average of the predictive weights for each predictor variable was calculated for the best final models.

Once trained, the network was applied to another random sample for validation. During this testing phase, the network does not receive the actual outcome information and performs the classification based on the models developed in the previous training phase, on a new vector matrix containing the predictor information for a different sample of children. In order to evaluate the performance of each model, the final confusion matrix for each one was determined during both phases. Values and rates for true positive (TP, in this study, low 33% of EC), true negative (TN, moderate/high-EC), false positive (FP), and false negative (FN) were calculated. Other quality measures were obtained including precision and recall or sensitivity, and both were given equal weight. Additionally, specificity and an F-1 score were calculated.

Finally, a sensitivity analysis was carried out for each ANN to provide a measure of the relative importance of each predictor. This method calculates how the output of the ANN changes according to modifications in that predictor while the remaining inputs remain fixed. 

Table 3 shows the topology of the ANN models developed and the architecture for each final model classification between low 33% EC and moderate/high EC.

## 3. Results

The descriptive measures for each predictive and target variable are presented in Table 4.

Table 5 shows the quality measures used to evaluate each model. ANN models using both attentional and environmental variables as input were able to identify 100% of the children belonging to both low EC and moderate/high EC groups. Therefore, the more inclusive models obtained a higher sensitivity and specificity, compared to those ANNs which involved either only attentional or environmental predictors. The final models using only attentional inputs achieved good sensitivity and correctly classified 75% of low-EC children. These attentional models produced very accurate classifications of those children who did not have low EC. Finally, models including only environmental factors were not able to correctly classify both groups of children simultaneously, achieving only relatively low accuracy values for both groups.

**Figure 4:**
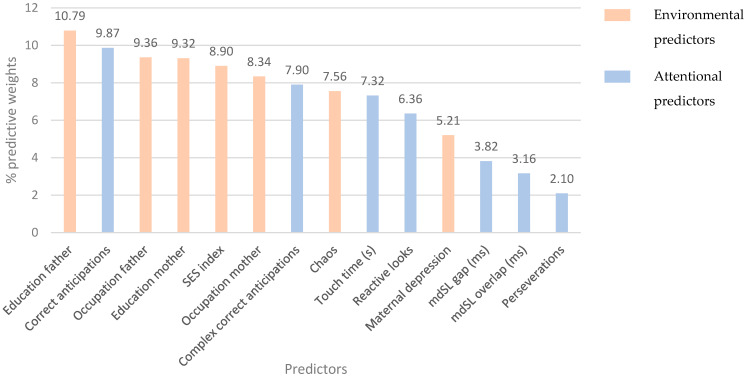
Predictive weights of the variables participating in the best model for the predictive classification of low EC.

Table 6 shows the importance of the classification of the predictor variables (factors and covariates) for each set of ANNs. Actual predictive weights of each predictor for the best model are presented in Figure 4.

Father’s education and correct anticipations were the top two predictors with the most significant importance in classifying between low EC vs. moderate/high EC. Furthermore, the inclusive model was able to correctly identify both groups, considering an interaction among attentional and other socio-economic variables such as the education of the mother, SES, father’s and mother’s occupation, and complex correct anticipations. These predictors contributed more than 60% of the predictive weight of the variables for reaching a correct predictive classification. However, it is important to observe that all variables contribute to the prediction in relatively small proportions, and it is the joint effect of many contributing variables that influences EC development.

## 4. Discussion

The main objective of this study was to identify children with low EC at 36 months old using predictive models considering attentional and environmental variables from early infancy (6 to 9 months of age). We compared three types of ANN models using (1) only attentional predictors, (2) only environmental predictors, and (3) both attentional and environmental predictors. 

The results show that it is possible to predict low EC at 36 months using data from as early as 6–9 months old, taking into account cognitive as well as environmental variables. However, there are differences in the accuracy achieved among the ANN models. The maximum accuracy in finding the target group was achieved when the ANN included both attention and environmental variables. This combined model was able to correctly classify low EC children (below percentile 33 of EC score) vs. moderate/high EC of the sample without any errors. On the other hand, when we considered only attentional measures from infancy, the model was able to correctly identify only 75% of the children with low EC. Finally, the models involving only environmental predictors achieved a lower level of accuracy in the identification of the target group (approximately only 50% of the children with low EC were identified). The higher accuracy of an attentional-only model compared with the environmental-only one supports the important role of attention in self-regulation development which has been demonstrated by extensive research in this field [18,85]. Attention has been proposed as the foundation for the development of EC [16,17,79]. In addition, attention and EC are key aspects in the development of self-regulation [21].

This study shows that the best predictive model of EC involved both attentional and environmental variables. This is consistent with the notion of self-regulation development as a complex process consisting of nonlinear relationships among individual attentional variables and the environment [12,35]. The ANN methodology has the advantage of capturing complex and nonlinear relationships among these early variables which seem to be indicators of a lower level of self-regulated behavior at a later age, even when there were no significant differences in individual predictors between the children at risk and moderate/high EC. The evaluation measures of the ANN in this study are consistent with previous research indicating their robustness for modeling complex patterns among variables associated with self-regulation and educational outcomes [65,86,87,88,89,90,91].

Among the early attentional variables in this study, those related to anticipatory attention in the VSL task (i.e., correct anticipations and complex correct anticipations) were the two strongest predictors. However, exogenous attention measured in the same task (i.e., reactive looks) also accounted for a smaller weight in the model. This is consistent with the developmental trajectory of attention. Exogenous attention is especially important from birth up to 3 months of age, when attention is mostly exogenously controlled by parents using external stimulation (i.e., shaking a rattle [85]). From this age onwards, volitional control experiences significant increases [92], accounting for the majority of improvements in infants’ attentional abilities [93]. Anticipatory attention between 4 and 6 months of age has been positively associated with self-regulated behavior (i.e., soothability [25,28]). Furthermore, this relationship is maintained during early childhood, with 30-month-olds’ correct anticipations in complex sequences being positively associated with EC [14]. The high importance found for variables related to endogenous control suggests that the development of the fronto-parietal network [94], and attentional processes associated with it, drives much of the predictive power of later self-regulatory abilities.

Infants’ capacity for self-restraint also had an important weighting in the model’s prediction. The ability to avoid touching an interesting object in the self-restraint task is a good measure for global inhibition in infants and toddlers, which is when the child is able to avoid an explicit behavior without being required to perform an alternative one [76]. In this sense, the ability to engage inhibitory control is crucial for efficient self-regulated behavior [95] and is related to more executive control of attention and effortful behavior [29], contributing to children’s socio-emotional well-being and schooling competence [1].

Visual disengagement is of great importance in the first years of life, allowing infants to voluntarily orient their attention in the visual space [25]. The attention-only model seems to capture this importance on the later emergence of EC, as visual disengagement, especially in the overlap condition, has been positively associated with EC starting from 12 months of age [27]. However, once we accounted for interactions between attention and environment, it experiences a reduction in its importance. 

Perseverations had relatively small weights in both the attention-only and the attention-environment models. This result is likely to be related to the developmental trajectory of perseverative behavior. At around 6 months of age, infants have been found to display a low number of perseverations, as they are not able to form stable traces of visual representations in memory [96]. Perseverations increase towards the end of the first year of life [96,97], as a consequence of an improvement in the stability of their visual representations. Finally, during toddlerhood, perseveration decreases as a result of infants’ developmental gains in attentional flexibility [98]. The developmental trajectory of perseverative behavior could make perseverations a less appropriate predictor of later self-regulated behavior at 6 months of age, as the lower ability to form stable traces in memory leads to predominantly correct reaching [96].

Environmental predictors related to SES, specifically the father’s education and occupation as well the SES index, contributed with high predictive weights to the model that can identify children with low EC. These results fit with previous studies which found differential effects of SES on cognition during childhood from 4 to 11 years old [99,100]. Low-SES environments involve higher exposure to stress [101] and lower cognitive stimulation [102], impacting negatively on executive function development. However, the SES-executive functions relationship varies between low to medium in size depending on several moderators such as the SES variability in the sample, number, and methods used to measure EF, but it remains stable across childhood [103]. Although the sample in this study has a modest SES variability, the pattern of interaction effects between these early SES factors in the environment with cognitive markers of attentional functions resulted in a plausible model in the ANN analyses [91,104].

It is important to note that given the absence of statistically significant differences between the low-EC group and moderate/high-EC group, it is the pattern of interactions amongst all the participating variables in the vector of information of each child that captures the information necessary to achieve the degree of precision of each model. It is not surprising that adding the environmental variables to the attention-only model would increase the density of information and therefore produce a more effective and predictive model (especially taking into account that an environment-only model had already achieved 50% accuracy). Information theory and the holographic principle [105,106] already postulate this effect, with the notion that each information piece would contribute to the density, which in turn will increase the precision of a model [107]. Of course, the shorter the distance and the more closely related a variable is to the desired effect to be measured (low EC in this case), the greater its weight, and greater precision can be achieved with a lower density than would be required from variables more distantly related.

Regarding chaos, early exposure to a disorganized and unpredictable household seems to have a moderate weighting on the prediction of children’s EC levels. Previous studies have found higher levels of chaos to be related to lower EC [77], EF [49], and self-regulated behavior [50]. Our model captures the importance of home chaos, although the increase in the predictive weight of this variable from the environment-only model to the attentional-environment model suggests an important interaction with attentional abilities that also contribute to a better classification.

Contrary to chaos, the predictive weight of maternal depression is reduced when accounting for attention variables. This is interesting, as previous research has found maternal depression to negatively impact infants’ negative affectivity [108], as well as the emergence of EF [58,59] and behavioral problems [109]. This indicates that babies’ attentional capacities could act as a protective factor against the impact of caregivers’ dispositional conditions. Nevertheless, maternal depression continues to have a moderate weight in the combined predictive model, which is in line with the mentioned literature.

This study has several limitations. Firstly, we only used parents’ reported measures of children’s EC at 36 months. Although this temperamental factor is a robust predictor of the development of self-regulation and a set of life outcomes, including academic achievement and socio-emotional adjustment, along with development [16,110], it would be good to include objective measures of self-regulated behavior in future studies. Secondly, although we have included relevant factors to design a predictive model, there are more specific environmental variables that could contribute to the development of self-regulation such as language stimulation and parental styles that were not considered in the present study. It is also the case that other specific individual variables such as genetic factors were not included in this research. Therefore, the present machine-learning-based model should be considered as only one of the plausible models of the early development of self-regulation. Moreover, as more predictors are added to the model, the density of available information increases, which can lead to new plausible models that accurately categorize children’s early self-regulation characteristics. More research could take into account measures collected ecologically, available from large health and pre-school surveys, in order to earlier and faster detect typical and atypical trajectories of regulation. Thirdly, the sample size in this study was small and non-probabilistic. Although we validated the classification in the independent test set, given its limited size, it may still lack the representation of some different plausible patterns of early predictors. This potential lack of representativeness could have an impact on the generalizability of the relationships and patterns found in this sample. Future research is needed to replicate our findings in different and larger samples of children.

## 5. Conclusions

To sum up, the current study shows that the complex interactive pattern between early attention and environmental factors during infancy is able to provide a more accurate prediction of later EC abilities in early childhood. To the best of our knowledge, this study is the first research applying machine learning to predict self-regulated behavior in infants from early factors at 6 months of age. This is a relevant result, especially from an interventionist perspective. Our results support the notion that it is the complex interaction between cognition and environment that shapes infants’ development. Moreover, interactions between attention and environment are able to moderate the relative importance of factors. We have seen that certain variables experience changes in their predictive importance from the only-attention or only-environment to the attention-environment model. It should also be considered that the complex interactions between the attention and environmental factors considered in this study are only one plausible explanation for the early development of self-regulation.

## Figures and Tables

**Table 1:** Sample descriptive statistics.

	6 Months	9 Months	36 Months
*n*	142 (73 female)	122 (60 female)	92 (46 female)
Age (days)	193.80 (8.49)	284.75 (9.21)	1119.09 (18.42)
Weight at birth	3354.87 (472.43)	-	-
Gestational weeks	39.65 (1.38)	-	-

**Table 2:** Predictive variables measured by each task and questionnaires at 6 and 9 months of age and its associated construct.

Age of Measurement	Task/Questionnaire	Variable	Construct
6 months	Gap-overlap	mdSL overlap	Attentional disengagement
		mdSL gap	Attentional orienting
	Switching	Perseverations (post-switch)	Attentional flexibility
	VSL	Reactive looks	Reactive attention
		Correct anticipations	Anticipatory attention
9 months		Complex correct anticipations	Anticipatory attention and monitoring
	Toy prohibition	Touch time	Self-restraint
6 months	SES	SES index	Family general socioeconomic status
		Mother’s education	Parent’s education level
		Father’s education	
		Mother’s occupation	Parents’ occupational level
		Father’s occupation	
	CHAOS	Chaos	Household disorganization
	BDI	Maternal depression	Maternal depressive symptomatology
36 months	CBQ	Effortful Control *	Children’s self-regulated behavior

Note. * Denotes the target variable used in the ANN model. mdSL = Median Saccade Latency; VSL = Visual Sequence-Learning task; SES = Socioeconomic Status; CHAOS = Confusion, Hubbub, and Order Scale; BDI = Beck’s Depression Inventory; CBQ = Children’s Behavior Questionnaire.

**Table 3:** Architecture of ANNs.

Topology	Attention Model		Environment Model		Combined Model	
Training Set Data Testing Set Data	76.1%; *n* = 35	78.3%; *n* = 36	77.8%; *n* = 42	81.8%; *n* = 45	87.9%; *n* = 29	69.7%; *n* = 23
	23.9%; *n* = 11	21.7%; *n* = 10	22.2%; *n* = 12	18.2%; *n* = 10	12.1%; *n* = 4	30.3%; *n* = 10
Cross-entropy error Stopping error	15.243	10.073	15.888	12.397	0.015	0.146
	1 consecutive step with no decrease in error		1 consecutive step with no decrease in error		1 consecutive step with no decrease in error	
Number of input nodes	7		31	32	37	
Number of output units	2		2		2	
Number of hidden layers	1 hidden layer with 2 units		1 hidden layer with 1 unit		1 hidden layer with 5 units	1 hidden layer with 1 unit
Number of epochs for training	10		10		10	
Method for rescaling covariates	Standardized method		Standardized method		Standardized method	
Activation function for hidden layers	Hyperbolic tangent		Hyperbolic tangent		Hyperbolic tangent	
Activation and error function for output layer	Softmax. Cross-entropy.		Softmax. Cross-entropy		Softmax. Cross-entropy	
Methodology in the training phase	Online (one case by cycle)		Online		Online	
Parameters	Initial learning rate = 0.1 Momentum = 0.9 Optimization algorithm: gradient descent Minimum relative change in training error = 0.0001	Initial learning rate = 0.04 Momentum = 1.2 Optimization algorithm: gradient descent Minimum relative change in training error = 0.0001	Initial learning rate = 0.4 Momentum = 0.9 Optimization algorithm: gradient descent Minimum relative change in training error = 0.0001	Initial learning rate = 0.0004 Momentum = 1.5 Optimization algorithm: gradient descent Minimum relative change in training error = 0.0001	Initial learning rate = 0.6 Momentum = 0.7 Optimization algorithm: gradient descent Minimum relative change in training error = 0.0001	Initial learning rate = 0.8 Momentum = 0.5 Optimization algorithm: gradient descent Minimum relative change in training error = 0.0001

Note. The gradient descent optimization algorithm takes steps proportional to the negative of the approximate gradient of the function at the current point. Cross-entropy function accelerates the backpropagation algorithm, and it provides good overall network performance with relatively short stagnation periods.

**Table 4:** Mean and standard deviation descriptive statistics for the predictive and target variables measured for each task and questionnaire.

Task/Questionnaire	Variable	M (SD)
Gap-overlap	mdSL overlap (ms)	451.84 (100.40)
	mdSL gap (ms)	275.59 (30.18)
Switching	Perseverations (post-switch; %)	68.41 (34.14)
VSL	Reactive looks (%)	88.31 (11.19)
	Correct anticipations (%)	11.51 (10.89)
	Complex correct anticipations (%)	22.54 (27.50)
Toy prohibition	Touch time (s)	5.91 (6.38)
SES	SES index (z-score)	0.08 (0.82)
	Mother’s education	4.10 (1.54)
	Father’s education	3.49 (1.72)
	Mother’s occupation	3.88 (3.38)
	Father’s occupation	4.59 (2.73)
CHAOS	Chaos	41.09 (13.07)
BDI	Maternal depression	10.74 (7.43)
CBQ	Effortful control	4.91 (0.57)

Note. ms = milliseconds; s = seconds; mdSL = Median Saccade Latency; VSL = Visual Sequence-Learning task; SES = Socioeconomic Status; CHAOS = Confusion, Hubbub, and Order Scale; BDI = Beck’s Depression Inventory; CBQ = Children’s Behavior Questionnaire.

**Table 5:** Quality indicators of each model predicting low EC in the training and testing phases.

Measures	Attention Model				Environment Model				COMBINED MODEL			
	NN1		NN2		NN1		NN2		NN1		NN2	
	Train	Test	Train	Test	Train	Test	Train	Test	Train	Test	Train	Test
Accuracy for “Low-EC” group (TP): Sensitivity/Recall.	0.82	0.75	0.82	0.75	0.64	0.50	0.67	0.50	1	1	1	1
Accuracy for “moderate/high-EC” group (TN): Specificity.	0.79	1	0.88	1	1	1	0.97	0.83	1	1	1	1
Overall Accuracy	0.80	0.91	0.86	0.90	0.88	0.91	0.88	0.70	1	1	1	1
Precision	0.64	1	0.75	1	1	1	0.89	0.67	1	1	1	1
F1 score	0.72	0.86	0.78	0.86	0.78	0.67	0.76	0.57	1	1	1	1
AUC	0.87		0.96		0.75		0.93		1		1	

Note. TP = True Positives; FP = False Positives; FN = False Negatives; TN = True Negatives; AUC = Area Under the Curve. Sensitivity or recall (TP/(TP + FN)) represents the proportion of correctly identified targets out of all targets presented in the set. Specificity (TN/(TN + FP)) is the proportion of correctly identified non-targets out of all true-non-targets presented in the set. Precision (TP/(TP + FP)) represents the proportion of correctly identified targets out of all true targets presented to the system. The F1-Score (2TP/(2TP + FP + FN)) is the harmonic mean of Precision and Recall, taking both false positives and false negatives into account. The area under the ROC curve represents the true-positive rate (Sensitivity) plotted as a function of the false-positive rate (100—Specificity) for different cut-off points and it can be viewed as a measure of the overall model performance across all possible thresholds, that is, how well it distinguishes between two groups.

**Table 6:** Average importance of the variables participating in the three ANNs for the predictive classification of low EC.

Attentional Predictors	Importance	Environmental Predictors	Importance	Attentional and Environmental Predictors	Importance
Correct anticipations	0.19	Maternal depression	0.23	Education father	0.11
mdSL overlap (ms)	0.19	Education father	0.19	Correct anticipations	0.10
Reactive looks	0.18	Occupation father	0.17	Occupation father	0.09
mdSL gap (ms)	0.16	Occupation mother	0.14	Education mother	0.09
Perseverations	0.11	Education mother	0.14	SES index	0.09
Complex correct anticipations	0.10	Chaos	0.08	Occupation mother	0.08
Touch time (s)	0.07	SES index	0.06	Complex correct anticipations	0.08
				Chaos	0.08
				Touch time (s)	0.07
				Reactive looks	0.06
				Maternal depression	0.05
				mdSL gap (ms)	0.04
				mdSL overlap (ms)	0.03
				Perseverations	0.02

Note. The variables are arranged in decreasing order of importance for the predictive classification in each ANN.

## Data Availability

The data presented in this study are available from the corresponding author upon request.

## Supplementary Materials

The following supporting information can be downloaded at: https://www.mdpi.com/article/10.3390/children10060982/s1, Additional information about experimental tasks (gap-overlap, visual sequence learning, switching and toy prohibition tasks). References [111,112] are also cited in Supplementary Materials.
