# Supplementary material for: Predicting Effortful Control at 3 Years of Age from Measures of Attention and Home Environment in Infancy: A Machine Learning Approach
Source: Children (Basel). 2023 May 31;10(6):982. doi: 10.3390/children10060982 (PMC10297630; doi:10.3390/children10060982)
Supplement: Supplementary file 1 [file children-10-00982-s001.zip › children-2379949-supplementary.pdf]

# Predicting Effortful Control at 3 Years of Age from Measures of Attention and Home Environment in Infancy: A Machine Learning Approach

## Supplementary Materials

### 1 Experimental tasks supplementary information

*1.1. Gap-overlap task*

Central and peripheral stimuli were randomly selected from a pool of 74 and 6 stimuli for central and peripheral stimulus, respectively. In order for the trial to be considered valid, infants had to look at the central stimulus during the last 200 ms before the peripheral target presentation, otherwise it was considered invalid and removed from analysis (Holmboe et al., 2018).

Two 16.34º x 20.47º areas of interest (AOIs) were created for the peripheral targets, while a 15.4º x 20.47º AOI was generated for the central stimulus. Saccade latencies (SL) were computed on valid trials subtracting the onset of the first fixation on the peripheral target from its onset. SL below the threshold of 120 ms were considered anticipatory and removed from analysis (Csibra et al., 2001). Infants were removed from subsequent analyses if: 1. Did not achieve a minimum of 4 valid trials in each experimental condition and 2. Experienced family interference during task administration or 3. Did not have enough data for pre-processing steps. A total of 42 infants were excluded from statistical analyses ($n$ = 25 for criteria 1; $n$ = 3 by criteria 2 and $n$ = 14 by criteria 3 at 6 months).

*1.2. Visual Sequence Learning (VSL) task*

1.2.1. Six-month-olds version

Two 19.02º x 26.56º AOIs were defined around each of the possible stimulus locations. In order to be considered for statistical analysis, infants were required to accomplish more than 50% of trials with stimulus fixations in practice and experimental trials (Rothbart et al., 2003). A total of 42 infants were excluded due to not meeting the experimental criteria of trials with stimulus fixations ($n$ = 39) or due to parent interference ($n$ = 3)

1.2.2. Nine-month-olds version

Two 19.02º x 26.56º AOIs were defined around each stimulus position in order to compute stimuli fixations, reactive looks and anticipatory looks. Only those trials in which an overt orienting of visual attention was allowed were considered to compute stimulus fixations, reactive and anticipatory looks, that is in trials in which attention could be oriented from position 1 to position 2, and from position 2 to position 1. It should be noted that in trials in which position 1 is again followed by position 1, infants were likely to remain fixed in that position during the anticipatory and reactive period. In these trials, the distinction of these two types of looks is hampered due to this sequence configuration. Considering all trials would lead to a lower number of reactive and anticipatory looks compared to the other versions of the task in which these events are allowed in all trials. Consequently, we decided to correct this difference between versions calculating these variables considering only these trials. The same exclusion criteria considered for the 6-mo-olds version were

applied. A total of 34 infants were excluded due to not meeting trials requirements criterion (*n* = 28) parent interference (*n* = 2) or infant cry (*n* = 4).

### 1.3. *Switching task*

Two 19.57º x 27.4º AOIs were generated for the left and right side of the screen (Conejero & Rueda, 2018). We excluded all fixations that occurred in the first 200 ms of the anticipatory period, as they do not reflect a real expectation, considering instead the first 200 ms of the stimulus presentation as part of the anticipatory period (Canfield & Haith, 1991). All anticipatory fixations were required to be followed by a fixation on the stimulus as a form to ensure that the infant was engaged in the trial, avoiding to code artifactual anticipations (i.e., coding an anticipation when the infant was moving gaze outside of the screen). If anticipations to the correct and incorrect side were recorded on the same trial, only the anticipatory fixation with the highest duration was considered (Kòvacs & Mehler, 2009). Infants were excluded from these analyses if they: 1. Displayed a tendency to anticipate to the non-rewarded location in the pre-switch block (*n* = 16); 2. Experienced family interference during task administration (*n* = 2); 3. Cried during task administration (*n* = 1) or 4. Did not achieve a minimum of 3 correct anticipations during the pre-switch block (*n* = 39).

### 1.4. *Toy prohibition task*

An attractive toy (a glitter wand with flashing-colored lights) that was previously out of sight, was grabbed and presented by the experimenter directing infant's attention towards it. Next, the experimenter turned on the lights of the glitter wand, holding it centrally in front of the infant but keeping it out of infant's reach, while saying "*[Infant's name] don't touch it. No*". Immediately after the instruction was delivered, the experimenter left the toy on the table in front of the infant within a reachable distance, turning his/her head to the side in order to look away from the infant. The glitter wand was left over a piece of slightly sticky material to avoid the glitter want to roll towards the infant. If the infant did not touch the glitter wand after 30 seconds, the experimenter encouraged the infant saying "*It's okay, you can touch it now*", "*You can touch it (pointing at the toy)*" after 35 seconds and finally "*Let's see what happens when we touch it*" after 40 seconds. The task was ended if the infant did not touch it after 45 seconds.
